# Comparison between RT-qPCR for SARS-CoV-2 and expanded triage in sputum of symptomatic and asymptomatic COVID-19 subjects in Ecuador

**DOI:** 10.1186/s12879-021-06272-8

**Published:** 2021-06-12

**Authors:** Ariel Torres, Martha Fors, Tamaris Rivero, Karina Pantoja, Santiago Ballaz

**Affiliations:** 1 Medicina General Integral, Máster en Enfermedades Infecciosas, Hospital Gineco-obstétrico Nueva Aurora Luz Elena Arismendi, Quito, Ecuador; 2 Escuela de Medicina, Facultad de Ciencias de la Salud, Redondel del Ciclista, Antigua Via a Nayón, Universidad de las Américas, 170125 Quito, Ecuador; 3 Medicina General Integral, Hospital General Santo Domingo de los Tsáchilas, Santo Domingo, Ecuador; 4 Enfermería, Hospital General Santo Domingo de los Tsáchilas, Santo Domingo, Ecuador; 5 Escuela de Ciencias Biológicas e Ingeniería, Universidad Yachay Tech, Hacienda San José s/n, San Miguel de Urcuquí, Ibarra, Ecuador; 6 Facultad de Ciencias Médicas Enrique Ortega Moreira, Universidad Espíritu Santo, Samborondón, Ecuador

**Keywords:** COVID-19, **RT-qPCR**, **SARS-CoV-2;sensitivity**, **Specificity**

## Abstract

**Background:**

The quantitative reverse transcriptase-polymerase chain reaction (RT-qPCR) effectively detects the SARS-COV-2 virus. SARS-CoV-2 Nevertheless, some critical gaps remain in the identification and monitoring of asymptomatic people.

**Methods:**

This retrospective study included 733 asymptomatic and symptomatic COVID-19 subjects, who were submitted to the RT-qPCR test. The objective was to assess the efficacy of an expanded triage of subjects undergoing the RT-qPCR test for SARS-COV-2 to identify the largest possible number of COVID-19 cases in a hospital setting in Ecuador. SARS-CoV-2 Firstly, the sensitivity and specificity as well as the predictive values of an expanded triage method were calculated. In addition, the Kappa coefficient was also determined to assess the concordance between laboratory test results and the expanded triage.

**Results:**

Of a total of 733 sputum samples; 229 were RT-qPCR-positive (31.2%) and mortality rate reached 1.2%. Overall sensitivity and specificity were 86.0% (95% confidence interval: 81.0–90.0%) and 37.0% (95% confidence interval: 32.0–41.0%) respectively, with a diagnostic accuracy of 52.0% and a Kappa coefficient of 0.73. An association between the positivity of the test and its performance before 10 days was found.

**Conclusions:**

The clinical sensitivity for COVID-19 detection was within acceptable standards, but the specificity still fell below the values of reference. The lack of symptoms did not always mean to have a negative SARS-COV-2 RT-qPCR test. The expanded triage identified a still unnoticed percentage of asymptomatic subjects showing positive results for the SARS-COV-2 RT-qPCR test. The study also revealed a significant relationship between the number of RT-qPCR-positive cases and the performance of the molecular diagnosis within the first 10 days of COVID-19 in the symptomatic group**.**

## Introduction

SARS-CoV-2 On February 2020, the World Health Organization (WHO) officially declared the outbreak of the new COVID-19 disease [1], a public health emergency caused by the rapid transboundary propagation of the new SARS-COV-2 virus. Due to this pandemic, multiple health systems and economies collapsed, and many others where at threshold and Ecuador was not an exception. . On March 22, 2021 from the start of the pandemic Ecuador reported 312.598 COVID-19-infected people and 16.451 deaths because of SARS-COV-2 infection, with 0.12% of the population fully vaccinated [2]. In the country a total of 1.102.383 of RT-qPCR have been performed until March 2021 [3], and approximately 6.5% of the population has been tested. As long as a vaccine is not yet available for all the population in the country, the best strategy is a timely diagnosis of COVID-19 to track the chain of infections. Bearing in mind that all the phases of SARS-COV-2 infection (symptomatic, pre-symptomatic and asymptomatic phase), infected subjects have been proven to be contagious, the application of methods for early identification and optimizing the expanded triage of suspected COVID-19 patients is critical for reducing the number of infections, hospitalized people and mortality. SARS-CoV-2 SARS-CoV-2 Given the worldwide expansion of the COVID-19 pandemic, disease-detection testing capacity remains a critical priority [4].

Sometimes called “molecular photocopying”, the real-time reverse transcriptase-polymerase chain reaction or RT-qPCR test is so far considered the most reliable diagnostic method and a valuable weapon to detect the positive cases of SARS-COV-2 infection. Despite being an excellent technique for the diagnosis of symptomatic patients who suffer from the COVID-19 disease, its effectiveness in diagnosing asymptomatic people remains problematic [5]. Asymptomatic people are a risk to other people because they also transmit the disease, especially those subjects with mild symptoms, who do not go to obtain medical assistance.

There are subjects bearing SARS-COV-2 virus who are asymptomatic during the course of the infection, whilst in others it produces disease, pneumonia and a lethal acute respiratory distress syndrome requiring intensive care support. The rate of false-negative RT-qPCR tests (sensitivity) for SARS-COV-2 is very worrying and it is caused for different reasons, such as a bad or improper sampling, delayed time to analysis, inadequate sample storage, or depending whether a patient is tested when viral load is absent or below a detectable threshold (too early or too late) among others [6]. Individual differences in the body response to SARS-COV-2 infection is a reason for concern in the control of the infection chain and disease propagation. In the context of the epidemic in Ecuador, the goal of this work was to assess the performance of a expanded triage evaluation of COVID-19 patients when compared to the RT-qPCR test for SARS-COV-2 virus detection in terms of sensitivity and specificity.

## Methods

### Study design

A descriptive-correlating, retrospective, cross-sectional study based on measurements of diagnostic accuracy.

### Data collection

The database was downloaded from digital clinical records (VIEPI System, National Epidemiological Surveillance System of Ecuador) of patients of all ages, who underwent the RT-qPCR test (CDC 2019-Novel Coronavirus Real-Time RT-qPCR Diagnostic Panel upper and lower respiratory specimens) from March to August 2020 at the Santo Domingo General Hospital (Santo Domingo de los Tsáchilas, Ecuador). In the first stage, the patient sample was split asymptomatic and symptomatic based on the case definition/expanded triage, and after then into positive (sick call) or negative (healthy call) depending on the results of RT-qPCR for SARS-COV-2. In a second stage, based on the cut-off time of the molecular test, the symptomatic group was in turn divided into those diagnosed within the first 10 days of the onset of the symptoms and those diagnosed from the 11th day onward. The inclusion criteria were the following: (1) patients who underwent a RT-qPCR assay on a sputum sample and (2) the variables of interest that appeared correctly registered in the database. Those patients who underwent RT-qPCR on nasopharyngeal and urine samples and those showing test variables not under the scope of the study were excluded.

The triage method under analysis and the categorization of a patient as “symptomatic” were conducted according to the definition established by the National Directorate of Epidemiological Surveillance of Ecuador and described in the “Operational Guidelines for Response to Coronavirus COVID-19,” released on March 312,020. The selection criteria were the following: (1). A patient with acute respiratory illness (fever and at least one sign such as symptom of respiratory illness, for example, cough, shortness of breath), and a history of travel or residence in a country outside of Ecuador or to another town in Ecuador, reporting a transmission community from COVID-19 disease, during the 14 days before the onset of symptoms; (2) A patient with an acute respiratory disease who was in contact with a confirmed or probable COVID-19 case in the last 14 days before the onset of symptoms; (3) A patient with severe acute respiratory syndrome (fever and at least one symptom of respiratory illness, cough, shortness of breath requiring hospitalization) in the absence of an etiological diagnosis fully accounting for the clinical presentation [4].

Everyone at risk of being exposed to the virus (asymptomatic patients) were included in the expanded triage, like health personnel in close contact with symptomatic patients; RT-qPCR-positive patients; companions of symptomatic and RT-qPCR- positive patients having associated risk factors (e.g., mellitus diabetes, obesity, arterial hypertension, cancer, chronic pneumonia and elderly). In addition, subjects suffering from febrile syndrome with less than 24 h of evolution and having associated risk factors as well as patients having fever with no focal signs and either with or without associated risk factors.

### Sample size

No representative sample calculation was performed. The study only included a selection of 733 subjects who strictly met the selection criteria from the total number of patients who underwent a sputum RT-qPCR test at the Santo Domingo General Hospital from March to August 2020.

### Database description

The information obtained from patients were compiled into a single dataset with the following variables: age group, gender, health professionals (yes or no), comorbidities, condition (dead or alive), and date of the onset of symptoms (cut-off time: “Equal or less than 10 days” and “More than 10 days”) [4, 7].

### Statistics

All analyses were performed using the statistical SPSS software, version 24.0 for Windows (SPSS Inc., Chicago, IL). A first descriptive analysis of nature of the patient sample consisted of a relation of categorical variables expressed as absolute frequencies and proportions. Using RT-qPCR test outcomes as reference, symptomatic and asymptomatic patients were grouped in a 2 × 2 contingency table to calculate the sensitivity (true positives/sick calls), specificity (true negatives/healthy calls), positive predictive value or PPV (true positives/positive calls), and the negative predictive value or NPV (true negatives/negative calls). Confidence intervals were set at 95% for each of these indicators. Sensitivity was defined as the proportion of true positives over a combination of true positives and false negatives in the entire sample. Specificity was set as the proportion of true negatives over a combination of true negatives and false positives in the entire sample. Positive likelihood ratios (LR+) and negative likelihood ratios (LR-) were also calculated. The Cohen’s kappa coefficient with 95% confidence intervals were calculated to evaluate the level of concordance for RT-qPCR outcomes and clinical condition variables. Cohen’s kappa values were then categorized as follows [8]: poor (< 0.20), fair (0.21–0.40); moderate (0.41–0.60), good (0.61–0.80), very good (0.81–1.00). Additionally, a Chi-Square test was run in the symptomatic group to challenge the association between RT-qPCR test outcomes and before and after the cut-off time (10 days) of the expanded triage. A Two-sided *P*-value below 0.05 was considered statistically significant.

### Ethics

Written informed consent was waived due to the retrospective nature of the study. The study was approved by the Ethics Committee of the Santo Domingo General Hospital and conducted in accordance with the ethical policies established by Ecuadorian legislation (Public Health Ministerial order of December 31st, 2014). The authors declare they had no access to identifying patient information when analyzing the data. We followed the STROBE guideline to report this study.

## Results

### Sample characteristics

**Fig. 1:**
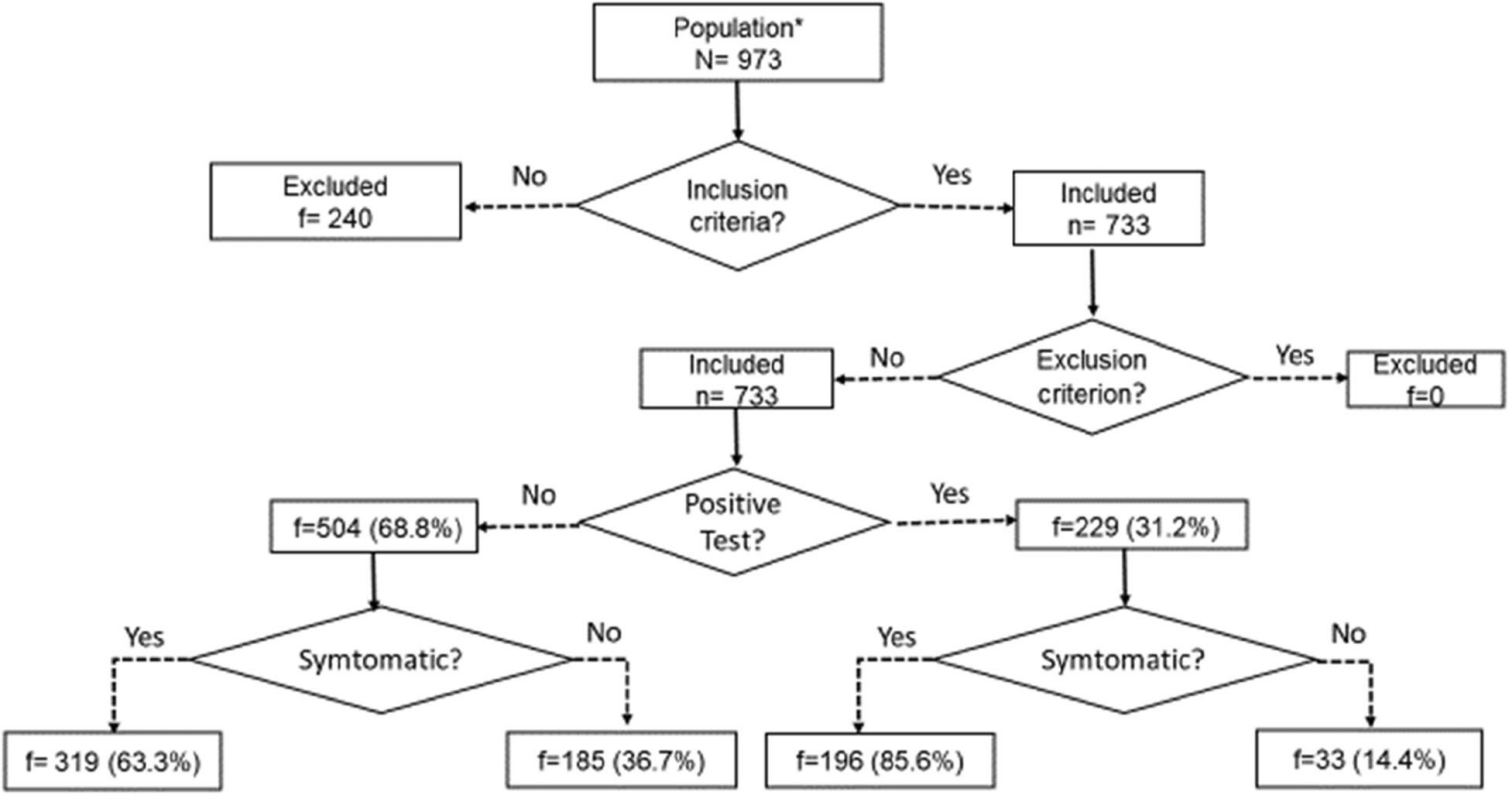
Patient flow diagram by final diagnosis and SARS-CoV-2 RT-PCR

A total of 733 clinical records who met the established criteria were considered for the analysis. Patients who underwent RT-qPCR on other samples (nasopharyngeal and urine), and those from whom it was not possible to obtain correct data corresponding to the variables of interest were excluded. A total of 229 patients (31.2%) were RT-qPCR-positive for SARS-COV-2. (Fig. 1).

The scrutiny of the clinical records of the 733 subjects included in this study revealed that the majority were outpatients in the range of 20–49 years old, approximately 9% were health professionals, the mortality rate reached 1.2%. SARS-CoV-2 (Table 1).

**Table 1:** Nature and distribution of the sample

	N	%
**Age group**		
1–14 years	38	5.2
15–19 years	33	4.5
20–49 years	461	62.9
50–64 years	127	17.3
> 65 years	74	10.1
**Gender**		
Women	389	53.1
Men	344	46.9
**Health professionals**		
No	668	91.1
Yes	65	8.9
**Comorbidities**		
No	689	94.0
Yes	44	6.0
**Type of care**		
Outpatients	654	89.2
Hospitalized	79	10.8
**Condition**		
Dead	9	1.2
Alive	724	98.8

Table 2 shows the results corresponding to the sensitivity and specificity of the RT-qPCR test. The percentage of symptomatic patients that according to the RT-qPCR test were positive for SARS-COV-2 (196 true positives out of 229 sick calls) was 86% (sensitivity). However, the percentage of subjects who were asymptomatic and whose RT-qPCR tests were negative for SARS-COV-2 relative to the total of patients with negative RT-qPCR results (185 true negative out of 504 healthy calls) dropped to 37% (specificity). Regarding the percentage of patients who gave a positive result in the RT-qPCR test among those who were actually symptomatic (196 sick calls out of 515 true positives) was 38% (PPV), whilst the percentage of patients who yielded negative results in the RT-qPCR test among those who looked healthy (185 healthy calls out of 218 true negatives) was 85% (NPV). Considering the results, the positive likelihood ratio (LR+) was above 1, and the negative likelihood ratio (LR-) approached to 0, thus indicating that the test showed reasonably good discriminatory power. It was revealed that 15.1% of the asymptomatic subjects were positive RT-qPCR cases. After correction for chance agreement, a Kappa value of 0.73 denoted a very good concordance between the outcomes of the molecular diagnostic test (positive vs. negative) and the expanded triage (asymptomatic vs. symptomatic).

**Table 2:** Assessment of the Expanded Triage against Diagnostic Test (RT-qPCR) in Sputum. (*n* = 733)

Condition	RT-qPCR SARS-CoV-2 Results		
	Positive	Negative	Total
Symptomatic subjects	196	319	515
Asymptomatic subjects	33	185	218
Total	229	504	733
Sensitivity	86% (95%IC:81–90)		
Specificity	37% (95%IC:32–41)		
Positive Predictive Value (PPV)	38% (95%IC:34–42)		
Negative Predictive Value (NPV)	85% (95%IC:80–90)		
Accuracy	52%		
Positive likelihood ratio	1.36		
Negative likelihood ratio	0.37		
Kappa coefficient	0.73 (95%IC:69–77)		

Finally, this study revealed a statistically significant relationship between the performance of the molecular diagnosis within the first 10 days of the progress of COVID-19 and the number of RT-qPCR-positive cases in the symptomatic group (Table 3).

**Table 3:** Correlation between the Molecular Diagnosis Time and RT-qPCR Test Outcome in symptomatic (*n* = 515) subjects

Time	RT-qPCR result n = 515		
	Positive	Negative	Total
> 10 days	22	57	79
≤ 10 days	174	262	436
Total	196	319	515
*p-value	0.04		

Chi-square test. X^2^ (1, 515) = 4.12, *p* = 0.04

## Discussion

This investigation evaluated the efficacy of an expanded triage for suspected COVID-19 patients in a sample of 733 subjects from a single medical center tested between March and 2020 in Ecuador. As an important issue we found that approximately 9% of the subjects in our sample were healthcare professionals who potentially experience greater risks [9]. The sensitivity or true positives rate of the RT-qPCR method analyzed in our study was 86% (Table 2). This sensitivity was slightly superior to that reported elsewhere, which varies from 79% in Wuhan (China) [10–12]. Nevertheless, the sensitivity herein presented (86%) was still far from the almost 100% sensitivity reported by the German Charité Institute of Virology [13]. A study with a sample of 193 patients in the Netherlands reported a sensitivity of 89.2%, whereas the specificity was 68.2%, [14], higher than in our study. A meta-analysis that grouped 16 studies showed that the highest sensitivity of the RT-qPCR test was found in sputum specimens with an average of 97.2% and a range from 90.3 to 99.7% [15]. Despite using sputum samples for the diagnosis of the COVID-19 disease in our study, the differences in sensitivity were likely be related to the specific guidelines and directives ruling COVID-19 diagnosis across countries.

Whereas the sensitivity (86%) was not far off the range reported (from 56 to 83%), that was not the case of the specificity (37%), which exceeds 95% in one consulted authoritative reference [16]. The RT-qPCR test only detects SARS-CoV-2 infection, but not of the symptomatology of COVID-19. The application of an expanded triage identified 15.1% of RT-qPCR-positive cases among the asymptomatic subjects. In the context of a community epidemic, and having in mind that the expanded triage considered the asymptomatic patients at risk of being exposed to the virus as healthy, it should be expected that the specificity fell below the values of reference.

A low specificity does not complicate clinical decision-making, as does the number of RT-qPCR-positive subjects who are asymptomatic. Most people infected with SARS-COV-2 have mild illness with nonspecific symptoms, whereas only about 5% of the patients become seriously ill with respiratory failure, septic shock and multiple organ failure. Nevertheless, an unknown percentage of infected individuals (approx. 80%) never experience symptoms of COVID-19. Asymptomatic and sometimes mild-to-moderate COVID-19 patients do not see a doctor, so that they cannot be diagnosed as positive for COVID-19, thus contributing to the large-scale community transmission. Being able to identify those patients with a high probability of COVID-19 despite a negative RT-qPCR test is crucial for an effective clinical care [16]. The PPV found in this study (38%) was much lower than a study from Italy, which reported 86.4% [17]. Our sample included patients who met the criteria to be defined as a “suspicious” case, as well as subjects having similar symptoms and sharing the focus of infection, but who ended up taking the diagnostic test when they were included in the expanded triage. Even if the PPV in our study was too low, the NPV (85%) was consistent with what is reported elsewhere [17]. . Although our study included different groups of asymptomatic patients, in the light of the Chi-Square test (see Table 3), that the number of positive the RT-qPCR tests among symptomatic patients within the first 10 days of the molecular diagnosis was significantly higher compared to those obtained from the 11th onward. This gives support to the association of the positivity of the molecular diagnostic test and the taken of the samples within the first 10 days of the onset of the symptoms. According to one study, the detection of the viral RNA occurs at a higher percentage (from 65.3 to 93.4%) in samples collected within the first nine days of the onset of the symptoms, compared to samples obtained from ten days onward [7]. Our results agreed with some findings reported in China, where the diagnostic performance of the RT-qPCR progressively decreases to the point that the serological tests had a higher frequency of positivity compared to the molecular test, specifically later on ten days of COVID-19 symptoms (81% vs 64%), It has been observed that, as the disease progresses in time, the probability of detecting viral particles in respiratory samples decreases progressively, especially after the 10th day [4].

### Concluding remarks

Despite the limitations of this study (it is a retrospective and single–center nature analyses), it demonstrated that the definition of COVID-19 case and expanded triage were adequate for the detection COVID-19 cases. The diagnostic sensitivity of the RT-qPCR test was like previous reports. Interestingly, the expanded triage not only showed a good performance in diagnosing among symptomatic patients, but it also contributed to identifying a larger number of positive RT-qPCR cases in asymptomatic patients. The performance of the RT-qPCR test within the first 10 days of the COVID-19 symptoms showed a significant concordance with the positive results of this diagnostic proof. In the fight against the epidemic in Ecuador, a thorough surveillance of suspicious COVID-19 cases, largely those with associated risk factors and those exposed to the virus as in this study, could limit contagion, facilitate therapeutic intervention, and reduce morbidity and mortality rates.

## Data Availability

The datasets used and/or analyzed during the current study available from the corresponding author on reasonable request.

## Abbreviations

CI: Confidence interval
Covid-19: Coronavirus disease 2019
NPV: Negative predictive value
PPV: Positive predictive value
RT-qPCR: Real-time (quantitative) reverse transcription polymerase chain reaction
SARS-CoV-2: Severe acute respiratory syndrome coronavirus 2
